# Rehabilitation services and related health databases, Japan

**DOI:** 10.2471/BLT.22.288174

**Published:** 2022-09-20

**Authors:** Kaori Yamaguchi, Yasuhiro Nakanishi, Viroj Tangcharoensathien, Makoto Kono, Yuichi Nishioka, Tatsuya Noda, Tomoaki Imamura, Manabu Akahane

**Affiliations:** a Department of Health and Welfare Services, National Institute of Public Health, 2-3-6 Minami, Wako, Saitama, 351-0197, Japan.; b International Health Policy Program, Ministry of Public Health, Nonthaburi, Thailand.; c School of Health Sciences, International University of Health and Welfare, Odawara, Japan.; d Department of Public Health, Health Management and Policy, Nara Medical University, Kashihara, Japan.

## Abstract

The demographic transition towards an ageing population and the epidemiological transition from communicable to noncommunicable diseases have increased the demand for rehabilitation services globally. The aims of this paper were to describe the integration of rehabilitation into the Japanese health system and to illustrate how health information systems containing real-world data can be used to improve rehabilitation services, especially for the ageing population of Japan. In addition, there is an overview of how evidence-informed rehabilitation policy is guided by the analysis of large Japanese health databases, such as: (i) the National Database of Health Insurance Claims and Specific Health Checkups; (ii) the long-term care insurance comprehensive database; and (iii) the Long-Term Care Information System for Evidence database. Especially since the 1990s, the integration of rehabilitation into the Japanese health system has been driven by the country’s ageing population and rehabilitation is today provided widely to an increasing number of older adults. General medical insurance in Japan covers acute and post-acute (or recovery) intensive rehabilitation. Long-term care insurance covers rehabilitation at long-term care institutions and community facilities for older adults with the goal of helping to maintain independence in an ageing population. The analysis of large health databases can be used to improve the management of rehabilitation care services and increase scientific knowledge as well as guide rehabilitation policy and practice. In particular, such analyses could help solve the current challenges of overtreatment and undertreatment by identifying strict criteria for determining who should receive long-term rehabilitation services.

## Résumé

Tant la transition démographique vers un vieillissement de la population que la transition épidémiologique des maladies transmissibles vers les maladies non transmissibles ont entraîné une augmentation de la demande en services de réadaptation dans le monde. Le présent document poursuit plusieurs objectifs: décrire l'intégration de la réadaptation dans le système de santé au Japon, et illustrer comment les systèmes de santé contenant des données réelles peuvent être utilisés en vue d'améliorer de tels services, en particulier pour une population nipponne vieillissante. En outre, il offre un aperçu de la manière dont la politique de réadaptation étayée par des faits s'inspire de l'analyse de vastes bases de données sanitaires japonaises, parmi lesquelles: (i) la base de données nationale des demandes de remboursement au titre de l’assurance-maladie et des bilans de santé spécifiques; (ii) la base de données complète de l'assurance pour les soins longue durée; et enfin, (iii) la base de données du système d'information relatif aux attestations de soins longue durée. Le vieillissement de la population a poussé le Japon à inclure la réadaptation dans son système de santé, surtout depuis les années 1990; aujourd'hui, un nombre croissant de personnes âgées ont aisément accès à des services de réadaptation. Au Japon, l'assurance-maladie globale prend en charge la réadaptation intensive aiguë et post-aiguë (ou de rétablissement). De son côté, l'assurance pour les soins longue durée couvre la réadaptation dans les établissements dédiés et les infrastructures collectives accueillant des personnes âgées, avec pour but de contribuer à préserver l'autonomie au sein d'une population vieillissante. L'analyse de vastes bases de données sanitaires peut favoriser une meilleure gestion des services de réadaptation et accroître les connaissances scientifiques, mais aussi orienter les politiques et pratiques en la matière. Ce type d'analyse peut surtout aider à s'attaquer aux enjeux actuels que représentent les traitements excessifs ou insuffisants, en identifiant des critères stricts permettant de déterminer qui doit faire l'objet d'une réadaptation sur le long terme.

## Resumen

La transición demográfica hacia el envejecimiento de la población y la transición epidemiológica de las enfermedades transmisibles a las no transmisibles han aumentado la demanda de servicios de rehabilitación en todo el mundo. Los objetivos de este artículo son describir la integración de la rehabilitación en el sistema sanitario japonés e ilustrar cómo los sistemas de información sanitaria que contienen datos del mundo real se pueden utilizar para mejorar los servicios de rehabilitación, en especial para la población que envejece en Japón. Además, se ofrece una visión general de cómo la política de rehabilitación fundamentada en la evidencia se guía por el análisis de las grandes bases de datos sanitarias japonesas, como: (i) la Base de Datos Nacional de Reclamaciones al Seguro de Enfermedad y Chequeos Médicos Específicos; (ii) la base de datos integral del seguro de cuidados de larga duración; y (iii) la base de datos del Sistema de Información de Cuidados de Larga Duración para la Evidencia. En particular, desde la década de 1990, la integración de la rehabilitación en el sistema sanitario japonés se ha visto impulsada por el envejecimiento de la población del país y, en la actualidad, la rehabilitación se ofrece de forma generalizada a una cantidad cada vez mayor de adultos mayores. El seguro médico general de Japón cubre la rehabilitación intensiva aguda y posaguda (o de recuperación). El seguro de cuidados de larga duración cubre la rehabilitación en instituciones de larga estancia y centros comunitarios para adultos mayores con el objetivo de ayudar a mantener la independencia en una población que envejece. El análisis de las grandes bases de datos sanitarias puede servir para mejorar la gestión de los servicios de atención a la rehabilitación y aumentar los conocimientos científicos, así como para orientar la política y la práctica de la rehabilitación. En concreto, estos análisis podrían ayudar a resolver los problemas actuales de sobretratamiento y subtratamiento, al identificar criterios estrictos para determinar quién debe recibir servicios de rehabilitación de larga duración.

## ملخص

أدى التحول الديموغرافي باتجاه شيخوخة السكان وكذلك التحول الوبائي من الأمراض السارية إلى الأمراض غير السارية إلى زيادة الحاجة لخدمات التأهيل على مستوى العالم. تتمثل أهداف هذه الورقة البحثية في شرح دمج التأهيل في النظام الصحي الياباني وتوضيح كيف يمكن استخدام نُظم المعلومات الصحية التي تحتوي على بيانات واقعية من أجل تحسين خدمات التأهيل لا سيما تلك الموجهة للسكان من كبار السن في اليابان. وعلاوة على ذلك، هناك نظرة عامة على كيفية توجيه سياسة التأهيل القائمة على الأدلة عن طريق تحليل قواعد البيانات الصحية الضخمة في اليابان مثل: (1) قاعدة البيانات الوطنية لمطالبات التأمين الصحي والفحوص الطبية؛ و(2) قاعدة البيانات الشاملة لتأمين الرعاية الصحية طويل الأجل؛ و(3) قاعدة بيانات نظام معلومات الرعاية الصحية طويل الأجل للأدلة. ومنذ تسعينيات القرن الماضي على وجه التحديد، كان دمج التأهيل في النظام الصحي الياباني يُعزى إلى شيخوخة السكان في البلاد، بينما يتم تقديم خدمات التأهيل في الوقت الحالي على نطاق واسع إلى عدد متزايد من الراشدين الأكبر سنًا. يغطي التأمين الصحي العام في اليابان التأهيل المكثف للحالات الحادة والرعاية اللازمة بعد التعرض لحالة حادة (أو الشفاء). يغطي التأمين الصحي طويل الأجل التأهيل في مؤسسات الرعاية طويلة الأجل والمرافق المجتمعية للراشدين الأكبر سنًا وذلك بهدف المساعدة على الاحتفاظ بالاستقلالية لدى فئة كبار السن. يمكن استخدام تحليل قواعد البيانات الصحية الضخمة بغرض تحسين عملية تدبير خدمات الرعاية التأهيلية وزيادة المعارف العلمية، بالإضافة إلى توجيه سياسة التأهيل وممارساتها. يمكن أن تساعد مثل هذه التحليلات على وجه الخصوص في حل التحديات القائمة المتعلقة بالإفراط في العلاج وقصور العلاج وذلك عن طريق وضع معايير صارمة لتحديد الأشخاص الواجب حصولهم على خدمات التأهيل طويلة الأجل.

## 摘要

人口老龄化的加重以及从传染病到非传染病的流行病学转变导致在全球范围内需要享受康复服务的人越来越多。本论文旨在描述康复服务被纳入日本卫生系统的情况,并说明如何使用包含真实世界数据的卫生信息系统来改善康复服务,特别是针对日本老年人的康复服务。此外,还概述了如何通过分析日本大型健康数据库来指导循证康复政策,例如:(i) 国家医疗保险索赔和特定健康检查数据库;(ii) 长期护理保险综合数据库;以及 (iii) 长期护理证据信息系统数据库。特别是,自 1990 年代以来,日本的人口老龄化加快了康复服务被纳入日本卫生系统的进程,如今,需要向越来越多的老年人广泛提供康复服务。日本的一般医疗保险涵盖了急性和急性期后(或恢复期)的强化康复治疗费用。长期护理保险涵盖了长期护理机构和社区机构的老年人康复治疗费用,目的是为了帮助老年人维持独立性。对大型健康数据库的分析可用于改进康复护理服务的管理,增加康复科学知识以及指导康复政策和实践。特别是,此类分析可以帮助解决过度治疗和治疗不足的问题,通过制定严格的标准,确定哪些患者应该接受长期康复服务。

## Резюме

Демографический переход к старению населения, а также эпидемиологический переход от инфекционных заболеваний к неинфекционным заболеваниям увеличили спрос на реабилитационные услуги в глобальном масштабе. Цель данной статьи состояла в том, чтобы описать интеграцию реабилитации в японскую систему здравоохранения, а также проиллюстрировать, как системы информации в здравоохранении, содержащие реальные данные, могут использоваться для улучшения реабилитационных услуг, особенно для стареющего населения Японии. Кроме того, есть обзор того, как научно обоснованная политика в области реабилитации опирается на анализ крупных японских баз данных о здоровье, а именно: (i) национальная база данных по требованиям медицинского страхования и конкретных медицинских осмотров; (ii) всесторонняя база данных по страховому обеспечению долгосрочной медицинской помощи; (iii) информационная система долгосрочной медицинской помощи для доказательной базы данных. Уже начиная с 1990-х годов интеграция реабилитации в японскую систему здравоохранения была обусловлена старением населения страны, поэтому сегодня реабилитация широко предоставляется все большему числу пожилых людей. Общее медицинское страхование в Японии покрывает экстренную и постэкстренную (или восстановительную) интенсивную реабилитацию. Страхование на случай долгосрочной медицинской помощи покрывает реабилитацию в учреждениях долгосрочной помощи и общественных учреждениях для пожилых людей с целью помочь стареющему населению сохранить свою независимость. Для улучшения управления службами реабилитационной помощи и расширения научных знаний, а также для руководства политикой и практикой в области реабилитации можно использовать анализ больших баз данных о здоровье. В частности, такой анализ мог бы помочь решить текущие проблемы избыточного и недостаточного лечения путем установления строгих критериев для определения того, кто должен получать долгосрочные реабилитационные услуги.

## Introduction

Member States of the United Nations are committed to sustainable development goals,^1^ which include universal health coverage as a key driver of health and well-being. In addition, rehabilitation has been recognized as an essential component of universal health coverage.^2^ The demand for rehabilitation services is increasing globally due to the demographic transition towards an ageing population and the epidemiological transition from communicable to noncommunicable diseases. In 2019, the World Health Organization (WHO) estimated that 2.41 billion people required rehabilitation services.^3^ However, such services are not widely available nor adequately funded, particularly in low- and middle-income countries.^4^

We describe how rehabilitation services were integrated into the Japanese health system and discuss the potential of new health information systems based on real-world data for developing evidence-informed rehabilitation policies and practices that will strengthen services in the country.

## Rehabilitation in Japan

### Integrating rehabilitation into the health system

**Fig. 1:**
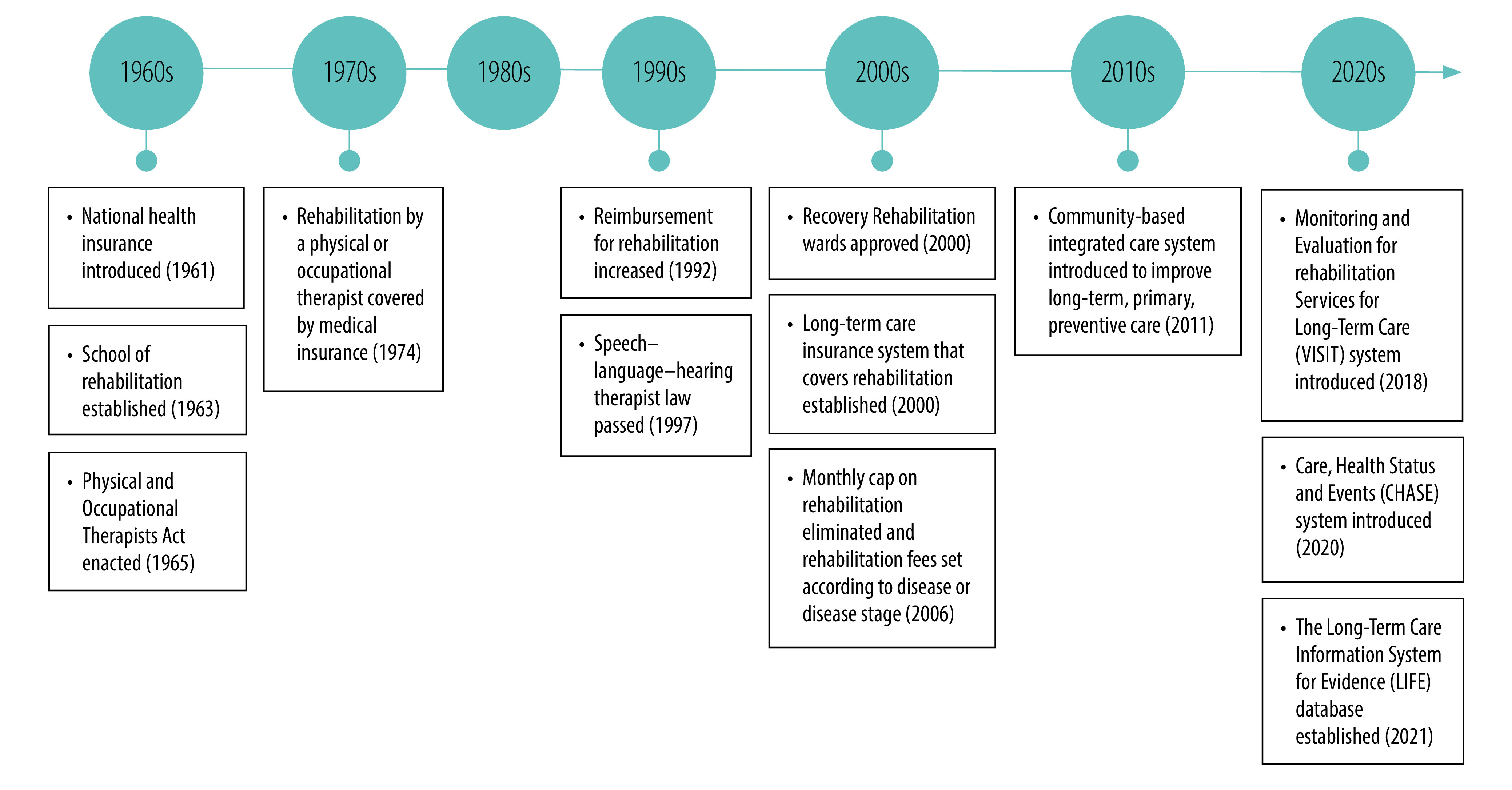
Integration of rehabilitation into the health system, Japan, 1961–2021

Historically, the Japanese government began to train rehabilitation professionals under the guidance of WHO. The first school of rehabilitation – the Professional School of Rehabilitation at the National Sanatoria Tokyo Hospital – was established in 1963 (Fig. 1), the Physical Therapists and Occupational Therapists Act was enacted in 1965 and the first national physical therapist and occupational therapist examinations were held in 1966.^5^^,^^6^ With the introduction of a speech–language–hearing therapist law in 1997, physical therapists, occupational therapists and speech–language–hearing therapists became established as the main rehabilitation professions in Japan. In 2021, there were 192 327 physical therapists, 94 255 occupational therapists and 36 255 speech–language–hearing therapists in the country.^7^^–^^9^ Because of its ageing population, today Japan has the highest rehabilitation workforce in the world and therapist-led rehabilitation is provided mainly within the health system.^5^

Japan achieved universal health coverage through the establishment of a national health insurance system in 1961.^10^^,^^11^ In 1974, reimbursement was revised and fees were established for rehabilitation services provided by physical and occupational therapists.^12^^,^^13^ In 1992, the importance of acute rehabilitation was recognized by, and promoted through, the health system and reimbursement for rehabilitation was increased substantially. In 2000, medical service fees were revised and a new type of hospital ward was approved to provide intensive rehabilitation after acute care: the recovery (i.e. convalescent or post-acute) rehabilitation ward. These wards are intended to provide sufficient rehabilitation for patients discharged from acute care hospitals to enable them to return home. Since 2000, an increasing number of rehabilitation professionals has been required for these new wards.^5^

A unit-based payment system for rehabilitation was introduced in 2002 under which a fee is charged for each 20-minute unit of rehabilitation. In 2006, a new payment system was introduced that stipulated time-limits on intensive rehabilitation for different disease groups.^5^ Although previously rehabilitation had been provided without regard to disease or disease stage, the new system divided diseases into two categories: (i) those requiring intensive rehabilitation; and (ii) those requiring nonintensive rehabilitation. Today, acute rehabilitation services are offered in many high-income countries. However, intensive rehabilitation in the post-acute phase is a particular characteristic of the Japanese system. The duration of intensive rehabilitation in Japan is until the maximum recovery possible.

Many high-income countries use a bundled payment system based on diagnosis-related groups to pay for rehabilitation services, whereas Japan employs a fee-for-service approach within the health insurance system.^14^ Although there are concerns about overtreatment with this approach, its intention is to enable older adults with disabilities to return home in the context of a country with a rapidly ageing population.

The long-term care insurance system established in 2000, which is based on the principle of supporting independent living, enables services such as nursing, long-term care and rehabilitation to be provided in both people’s homes and long-term care facilities.^15^ Under this system, health and care services (including rehabilitation) are available to people who have been certified as requiring support or long-term care (primarily older adults). Certification is based on the individual’s need for long-term care and is divided into seven levels, each of which specifies the range of services an individual can receive.^16^

In 2011, a community-based integrated care system was introduced following a revision of the Long-Term Care Insurance Law.^17^^,^^18^ The revised system was intended to enable older adults to spend the rest of their lives in their own neighbourhoods, even if they needed long-term care. The system places an emphasis on long-term, primary, preventive care, to which rehabilitation professionals are expected to contribute.^5^

### Current rehabilitation issues

Strict criteria need to be established for determining who should be covered by rehabilitation services because everyone’s health and well-being could potentially benefit from rehabilitation. After the intensive rehabilitation system was introduced in Japan in 2006, the maximum monthly limit on rehabilitation services was removed. Instead, the duration of intensive rehabilitation was determined for individual disease groups, which had the effect of counterbalancing supplier-induced demand for overtreatment to a certain extent. In addition, intensive rehabilitation was reserved for those diseases and clinical disease phases for which there was evidence that interventions were cost-effective and had substantial benefits for patients.

Long-term care insurance differs from medical insurance in having no clear criteria for determining which individuals who require long-term care can have access to rehabilitation. In extreme cases, patients may be permitted to undergo rehabilitation if they demand it, even if there is no real prospect of recovery. Another concern is overtreatment by providers but, on the other hand, there is also the possibility of undertreatment. For people who require long-term care, it might not be appropriate to set an initial limit to their rehabilitation based on disease duration or type because many other factors can contribute to functional decline and the resultant need for rehabilitation. In Japan, efforts have recently been made to improve the management of, for example, short-term intensive rehabilitation immediately after hospital discharge. Overall, it is vital that an effective system is established for providing long-term rehabilitation, especially for an ageing population.

## Real-world rehabilitation data

### Current databases

Globally, an increasing number of studies are making use of health-care databases.^19^^,^^20^ Administrative insurance claims databases, for example, contain a large amount of real-world data on the medical treatments and long-term care services covered by insurance. Retrospective studies using such data can complement the findings of randomized controlled trials and could even generate hypotheses for future trials.^21^ In addition, studies using real-world data can overcome some of the limitations of randomized controlled trials; for example, real-world data sets may include older patients and people with multimorbidities who are often excluded from trials. Large population studies can also be performed using real-world data and their findings may have greater external validity than those of randomized controlled trials. Additionally, the use of real-world data enables research to be conducted at a lower cost over a shorter time period and can be helpful for overcoming the lack of statistical power often encountered when studying rare events and diseases.^20^^,^^21^

**Fig. 2:**
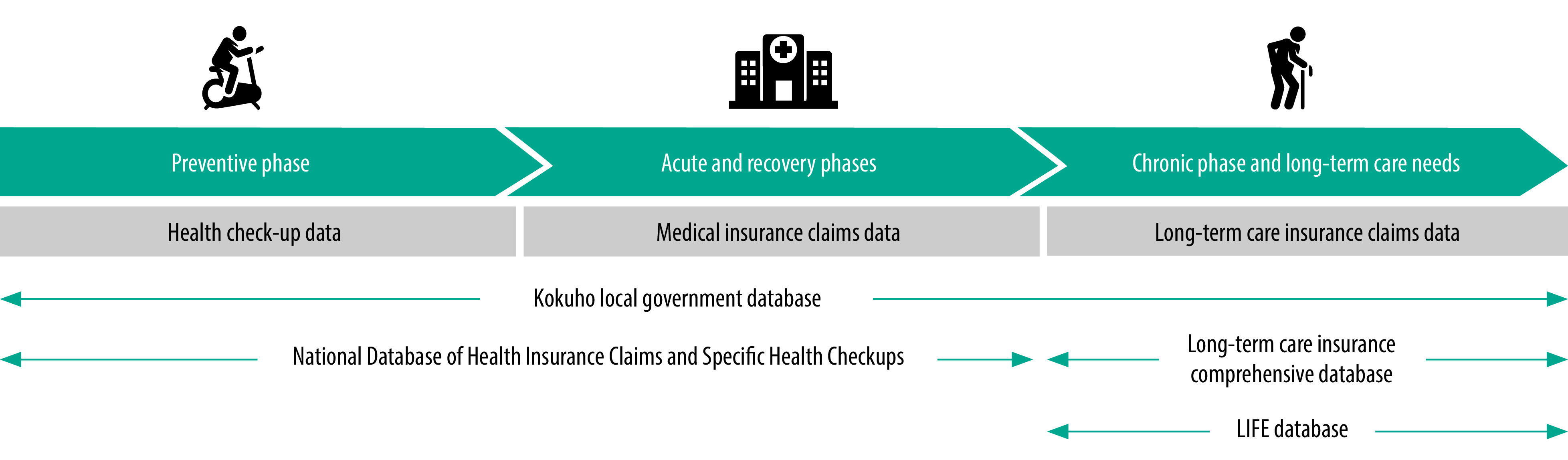
Rehabilitation data collected by four databases, by disease phase, Japan, 2022

The Japanese National Database of Health Insurance Claims and Specific Health Checkups is a comprehensive database covering insurance claims for medical service fees under Japan’s National Health Insurance system (Fig. 2).^22^ In 2022, it included information on approximately 22.5 billion claims from over 100 million individuals issued between April 2009 and December 2021, making it one of the world’s largest health-related databases.^22^^,^^23^ Since the Japanese government made the National Database available for research purposes in 2011, real-world data have been used for studies in fields such as health economics and clinical medicine. The Kokuho local government database, which includes insurance claims, has also been used for these purposes.^24^ The Kokuho database covers claims for both medical treatment and long-term care and some local governments link these data categories for individual patients. Consequently, individual patients can be followed from medical care to long-term care.^25^^,^^26^

LIFE: Long-Term Care Information System for Evidence.

Notes: The figure shows the rehabilitation data collected by four Japanese databases on individuals during different disease phases. In some prefectures, the Kokuho database contains both data from National Database of Health Insurance Claims and Specific Health Checkups and Long-term care insurance comprehensive databases if they are linked. Although the LIFE database and the long-term care insurance comprehensive database both cover the chronic phase and long-term care needs, they contain different information. For example, the LIFE database includes data on interventions and functional outcomes.

Although insurance claims data are particularly useful for descriptive epidemiological research and large-scale follow-up surveys, they are limited: the databases lack information on disease severity, on the socioeconomic characteristics of patients and their families, on the patients’ educational level and on laboratory test results, which are key outcome measures. This lack of information can confound study results, which therefore need to be interpreted with caution.^27^ Clinical studies should include outcome measures based on the tests used and treatments applied to compensate for the lack of outcome measures in claims data.

### Analysis of databases

Details of rehabilitation services provided under the medical insurance system are stored in the National Database of Health Insurance Claims and Specific Health Checkups, and the long-term care insurance comprehensive database contains information on rehabilitation provided both in the community and in long-term care facilities (Table 1).

**Table 1:** Databases containing rehabilitation data, Japan, 2022

Database characteristic	National Database of Health Insurance Claims and Specific Health Checkups	Long-term care insurance comprehensive database	Long-Term Care Information System for Evidence database
Target group	Whole population	People aged ≥ 65 years^a^ certificated as needing long-term care	People aged ≥ 65 years^a^ certificated as needing long-term care
No. of records	> 100 million^b^	7 million^c^	< 7 million^c^
Indicators of individual’s condition	None	Care needs level and information required for certification of care needs level^d^	Functional indicators, such as: (i) care needs level;^d^ (ii) Barthel Index; and (iii) performance of instrumental activities of daily living
Data entries relevant to rehabilitation	(i) Supply of acute and recovery rehabilitation by professionals for certain conditions, including cardiovascular, cerebrovascular, musculoskeletal and pulmonary disease, and disuse syndrome; (ii) supply of rehabilitation by professionals during all clinical phases of dementia, intractable disease and cancer, and for children or adults with disabilities	Supply of rehabilitation by professionals and functional training by trainers, and cooperative activities with rehabilitation professionals	Details of rehabilitation programmes provided by professionals and of functional training provided by trainers for each service user, reported using ICF categories (Box 1)

ICF: International Classification of Functioning, Disability and Health.

^a^ In addition, people aged 40 years or older with specified conditions (e.g. intractable disease) were included.

^b^ The 100 million people were those identified from different types of insurance claims data recorded between April 2009 and December 2020, such as medical inpatient claims, medical outpatient claims, diagnostic procedure combination claims (i.e. diagnosis-based claims) and pharmacy claims.^22^

^c^ The number of people certified as requiring long-term care or support in November 2021, as reported in public data from the Ministry of Health, Labour and Welfare.^28^

^d^ The care needs level was determined from the total estimated time needed for long-term care, which can be regarded as a functional indicator to some extent.

Previously the National Database was employed primarily to study the usage of rehabilitation services. For example, a recent study investigated cardiac rehabilitation for patients who underwent a percutaneous coronary intervention or coronary artery bypass grafting between April 2017 and March 2018.^29^ The study found that only one third of eligible patients participated in cardiac rehabilitation after treatment and that most underwent rehabilitation for too short a time. Another study that used linked medical and long-term care insurance claims administrative data sets investigated long-term care insurance beneficiaries who used home care services in Kashiwa city.^30^ This study included data on around 400 000 residents and reported an association between household income and the utilization of home-based rehabilitation and home help services. The study found that long-term care insurance beneficiaries with a low household income may forgo home-based rehabilitation and choose to minimize out-of-pocket payments on home care services.

Other potential applications of the National Database in Japan include analysing the quality and utilization of rehabilitation services by geographical region (e.g. health service area or municipality). By using these large data sets, researchers can estimate future demand for rehabilitation in each geographical region, thereby guiding the expansion of rehabilitation services in a way that minimizes regional gaps.

### COVID-19 pandemic

One example of the application of these large data sets was our analysis of the impact of the coronavirus disease 2019 (COVID-19) pandemic on rehabilitation services. We used data from the National Database of Health Insurance Claims and Specific Health Checkups and long-term care insurance claims data to compare rehabilitation service utilization before the pandemic in 2019 with that during the pandemic in 2020.^31^^,^^32^ Table 2 shows the number of reimbursement claims from patients without COVID-19 for acute and recovery rehabilitation from medical institutions for different disease groups in 2019 and 2020. We found that the number of reimbursement claims increased in every disease group, with an especially large increase among patients with pulmonary disease. However, the number of claims decreased among people aged under 40 years in all groups. Table 3 shows the number of claims in 2019 and 2020 from individuals covered by long-term care insurance for rehabilitation at home or in a day-care facility or for short-term intensive rehabilitation in long-term care facilities.^32^ While claims for rehabilitation at home increased, claims for day-care rehabilitation decreased across all age groups and for all levels of care needed. During the pandemic, day-care services, which ordinarily require older people to gather at facilities, were restricted to prevent the spread of infection. Overall, the magnitude of the increase in claims for rehabilitation at home was less than the magnitude of the decrease in claims for day-care rehabilitation, which suggests that some people might not have received rehabilitation because of the pandemic. In this way, the analysis of claims data can provide insights into the way rehabilitation services are dynamically responding during a pandemic.

**Table 2:** Insurance claims for acute and recovery rehabilitation from patients without COVID-19 before and during the pandemic, by disease group, Japan, 2019–2021

Claimant characteristic	Disease group														
	Cardiovascular disease			Cerebrovascular disease			Disuse syndrome			Musculoskeletal disease			Pulmonary disease		
	No. rehabilitation claims (thousands)		Difference in claim numbers between 2020 and 2019 (%)	No. rehabilitation claims (thousands)		Difference in claim numbers between 2020 and 2019 (%)	No. rehabilitation claims (thousands)		Difference in claim numbers between 2020 and 2019 (%)	No. rehabilitation claim (thousands)		Difference in claim numbers between 2020 and 2019 (%)	No. rehabilitation claims (thousands)		Difference in claim numbers between 2020 and 2019 (%)^b^
	2019^a^	2020^a^		2019^a^	2020^a^		2019^a^	2020^a^		2019^a^	2020^a^		2019^a^	2020^a^	
**All**	175 991	187 191	6.4	175 020	177 417	1.4	41 135	41 391	0.6	204 924	206 896	1.0	11 426	14 802	29.5
**Sex**															
Male	78 341	83 847	7.0	95 184	95 941	0.8	19 951	19 903	−0.2	63 741	65 524	2.8	6 714	8 861	32.0
Female	97 650	103 344	5.8	79 836	81 476	2.1	21 184	21 487	1.4	141 183	141 372	0.1	4 712	5 941	26.1
**Age, years**															
< 40	4 586	4 038	−12.0	11 867	10 642	−10.3	292	244	−16.3	10 780	10 031	−7.0	212	178	−15.8
40–64	23 100	22 497	−2.6	32 552	30 852	−5.2	2 401	2 293	−4.5	34 358	34 033	−0.9	745	784	5.3
65–74	35 056	35 627	1.6	39 718	38 799	−2.3	5 476	5 218	−4.7	40 053	38 399	−4.1	1 909	2 167	13.5
≥ 75	113 248	125 029	10.4	90 882	97 124	6.9	32 966	33 635	2.0	119 732	124 433	3.9	8 561	11 673	36.4

COVID-19: coronavirus disease 2019.

^a^ 2019 corresponds to the period between April 2019 and March 2020 before the coronavirus disease 2019 pandemic and 2020 corresponds to the period between April 2020 and March 2021 during the pandemic.^31^

^b^ Percentages were calculated from the original numbers and not from the rounded numbers reported in this table.

**Table 3:** Long-term care insurance claims for rehabilitation from patients without COVID-19 before and during the pandemic, by place of rehabilitation, Japan, 2019–2021

Claimants’ characteristic	Place of rehabilitation									
	In the community							In a long-term care facility (short-term intensive rehabilitation)^b^		
	At home^a^			In a day-care facility^a^						
	No. rehabilitation claims (thousands)		Difference in claims between 2020 and 2019 (%)^c^	No. rehabilitation claims (thousands)		Difference in claims between 2020 and 2019 (%)^c^		No. rehabilitation claims (thousands)		Difference in claims between 2020 and 2019 (%)^c^
	2019^d^	2020^d^		2019^d^	2020^d^			2019^d^	2020^d^	
**All**	1434	1499	4.6	7565	7080	−6.4		13 937	13 831	−0.8
**Age, years**										
40–64	115	117	2.4	310	283	−8.8		ND	ND	ND
65–74	271	280	3.1	1091	1019	−6.6		ND	ND	ND
≥ 75	1048	1103	5.2	6164	5779	−6.3		ND	ND	ND
**Level of support needed^e^**										
1	68	75	9.2	868	822	−5.3		ND	ND	ND
2	177	192	8.4	1292	1231	−4.8		ND	ND	ND
**Level of care needed^e^**										
1, lowest	242	263	8.7	1867	1766	−5.4		1 949	2 019	3.6
2	339	352	4.0	1790	1660	−7.3		2 885	2 853	−1.1
3	236	246	4.1	954	880	−7.8		3 473	3 467	−0.2
4	202	206	1.9	548	505	−8.0		3 770	3 708	−1.6
5, highest	169	164	−2.4	242	213	−12.0		1 860	1 784	−4.1

COVID-19: coronavirus disease 2019; ND: not determined.

^a^ The sum of the number of claims for rehabilitation at home and rehabilitation in a day-care facility is equal to the total number of rehabilitation claims for all individuals certified as requiring long-term care or support.

^b^ Short-term intensive rehabilitation included short-term intensive rehabilitation for dementia.

^c^ Percentages were calculated from the original numbers and not from the rounded numbers reported in this table.

^d^ 2019 corresponds to the period between April 2019 and March 2020 before the coronavirus disease 2019 pandemic and 2020 corresponds to the period between April 2020 and March 2021 during the pandemic.^32^

^e^ When individuals first require care, they apply for a certificate of long-term care, which entitles them to coverage by long-term care insurance. There are seven care needs levels: two support needs levels and five care needs levels. Care needs level 5 is the highest and represents the greatest need for care. The care needs level is determined from the total estimated time needed for long-term care.

In early 2022, with the appearance of the omicron variants of severe acute respiratory syndrome coronavirus 2, there was no sign of the pandemic ending and, by 13 January 2022, Japan had reported a total of 1.79 million cases and 18 412 deaths.^33^ Surveys conducted by WHO found that health systems globally were disrupted and unable to maintain essential health services for people without COVID-19 during the surge of infections.^34^^,^^35^ Moreover, other studies found that the pandemic had led to declines in activities of daily living, instrumental activities of daily living and cognitive functioning in older adults, as well as an increase in the incidence of depression.^36^^–^^38^ Researchers have also reported that the government’s pandemic containment measures resulted in an increase in the proportion of older adults who were frail.^39^ Clearly rehabilitation is important for maintaining and improving the functioning and capacity of older people. Worldwide, therefore, there is an urgent need to establish a system for providing rehabilitation.

The collection of good-quality evidence is essential for providing effective and efficient rehabilitation in a range of contexts, such as during the COVID-19 pandemic. However, there is a lack of evidence on the effectiveness of long-term rehabilitation.

## Long-term care database

The quality of long-term care has recently been recognized as important for dealing with issues that arise from population ageing. Consequently, there is a growing interest in Japan in developing evidence-based care on the same principles as evidence-based medicine.^40^ As a result, two new information systems were introduced in Japan: (i) the Monitoring and Evaluation for Rehabilitation Services for Long-Term Care (VISIT) system in 2018; and (ii) the Care, Health Status and Events (CHASE) system in 2020.^41^ In April 2021, a new national information system was established by central government with the aim of supporting independent living and preventing increases in the level of care needed: the Information System for Evidence (LIFE) database. The LIFE database aggregates information from VISIT and CHASE and contains real-world data on interventions and outcomes (particularly functional outcomes) in users of long-term care services. It is expected that evidence generated using the LIFE database will further the aims of government policy by helping to improve the performance and outcomes of long-term care services (including rehabilitation) and by assisting in the planning, implementation, monitoring and revision of long-term care insurance businesses.^41^

The LIFE database currently contains data submitted voluntarily by service providers on service users and on the services provided. In contrast, the National Database of Health Insurance Claims and Specific Health Checkups and the long-term care insurance comprehensive database cover the services provided by all agencies throughout the country. Data are submitted to the LIFE database when a reimbursement claim for long-term care is made, with each reimbursement claim corresponding to one long-term care service user. According to the August 2021 monthly report, 1275 million claims, which included information on users’ conditions, were submitted that month to the LIFE database from long-term care service providers.^32^ Notably, the number of claims does not equal the number of service users registered in the database because, for example, a user living at home may have received services from more than one provider and each provider may have submitted a separate claim for that user. Data in the LIFE database are anonymized before being stored on the national data server such that only service providers can identify individuals, thus ensuring confidentiality.

Recently, the government has started to use the LIFE database to help service providers improve the management of their businesses and service performance. Based on its analyses, the government gives providers feedback on each service user’s condition and the services performed so they can compare themselves against the national average and improve their operations if necessary. In addition, service providers are expected to enter details of changes in service users’ conditions into the LIFE database, which will also help improve the quality of services.

### Granularity of LIFE data

In addition to its use for improving the business management of care services, the analysis of LIFE data is expected to help guide policy and increase scientific knowledge. The LIFE database contains a wealth of information on individuals and their conditions and on rehabilitation interventions and their outcomes (Table 1 and Box 1), including sex, age, height, weight and the level of care required, as well as background information on, for example, diagnoses based on the International Statistical Classification of Diseases and Related Health Problems (10th revision), medical history, medication use and family structure. Details of each individual’s condition include, for example, their Barthel Index (a measure of activities of daily living), a Dementia Behaviour Disturbance Scale score, a nutritional status index and a decubitus scale score (a measure of the risk of pressure ulcers). The Barthel Index is commonly used to assess the clinical outcomes of rehabilitation services, especially for conditions such as stroke.^42^^–^^46^ When rehabilitation or functional training has been provided, information on the intervention is recorded and submitted to the LIFE database using the categories of the International Classification of Functioning, Disability and Health (e.g. muscle strength, eating and drinking ability, and cleaning living areas). This information is updated regularly and longitudinal data are produced for each individual. Consequently, the range of information contained in the LIFE database complements that provided by medical and long-term care claims data, thereby overcoming some of their limitations. As a result, the scope of research using real-world data in Japan could be broadened.

Box 1 **Categories used to report rehabilitation and functional training programmes in the LIFE database, Japan, 2022**

**Body functions and structures**

Confidence; visuospatial perception; language; mental functioning in sequencing complex movements; hearing; pain; voice; respiration; exercise tolerance functions; ingestion functions; joint mobility; muscle power; muscle tone; muscle endurance functions; and movement functions.

**Activities and participation**

Basic learning; learning to read; learning to write; learning to calculate; solving problems; making decisions; carrying out daily routine; handling stress and other psychological demands; changing basic body position; maintaining body position; transferring oneself; carrying, moving and handling objects; walking and moving; using transportation; washing oneself; caring for body parts; toileting; dressing; eating and drinking; looking after one’s health; acquisition of goods and services; preparation of meals; washing and drying clothes and garments; cleaning cooking area and utensils; cleaning living area; household tasks, other specified and unspecified; maintaining dwelling and furnishings; maintaining domestic appliances; taking care of plants; taking care of animals; general interpersonal interactions; remunerative employment; recreation and leisure; and products and technology.

ICF: International Classification of Functioning, Disability and Health; LIFE: Long-Term Care Information System for Evidence.Notes: Body functions, body structures, activities and participation are domains defined in ICF. Whenever rehabilitation or functional training is provided, information on the intervention is recorded in the LIFE database using the same categories as the ICF with the exception of two categories that are included in the LIFE database only: “giving information” and “teaching how to care.”

### Policy and practice

Today, it is common practice in Japan for care policy and practice to be guided by the evidence through knowledge translation. For instance, recently a long-term care prevention programme using a population approach was developed and adopted as national policy on the basis of the scientific evidence.^47^ In the near future, research findings based on LIFE data could be translated into policy. For example, insights gained from the analysis of LIFE data could lead to a revision of long-term care reimbursement fee schedules. If a LIFE-based data analysis identified effective rehabilitation interventions and appropriate targets for intervention, the introduction of incentives for effective interventions (e.g. additional fees) could result in better outcomes. Moreover, as little is known about the clinical effects of rehabilitation in older adults with chronic conditions or long-term care needs, the analysis of LIFE data could lead to major breakthroughs.

## Conclusion

We have summarized how rehabilitation was integrated into the Japanese health system and how large data sets can be used to develop evidence-informed rehabilitation policy and practice. Since the 1990s, in particular, the integration of rehabilitation has been driven by Japan’s ageing population and, today, a growing number of older adults are able to maintain their independence with the help of rehabilitation. The analysis of large data sets could also help solve current issues, such as overtreatment, by identifying strict criteria for determining who should receive long-term rehabilitation services.
